# Time course of changes in oxidative stress and stress-induced proteins in cardiomyocytes exposed to doxorubicin and prevention by vitamin C

**DOI:** 10.1371/journal.pone.0179452

**Published:** 2017-07-05

**Authors:** Ana Ludke, Gauri Akolkar, Prathapan Ayyappan, Anita K. Sharma, Pawan K. Singal

**Affiliations:** Institute of Cardiovascular Sciences, St Boniface Hospital Albrechtsen Research Centre, Department of Physiology and Pathophysiology, Max Rady College of Medicine, Rady Faculty of Health Sciences, University of Manitoba, Winnipeg, Canada; University of Central Florida, UNITED STATES

## Abstract

We previously reported that Vitamin C (Vit C) protects against doxorubicin (Dox)-induced cardiotoxicity by reducing oxidative stress, p38 mitogen-activated kinase (MAPK) and p53 activation and rescuing cell death in isolated adult cardiomyocytes. The pattern of activation and the role of oxidative stress as well as down-stream mechanisms for such protection remain elusive. Therefore the present study aims to analyze time-dependant generation of reactive oxygen species (ROS) and the activation of stress induced signalling pathways in cardiomyocytes treated with Dox and Vit C. The data provides further understanding of heart pathophysiology in response to Dox at the cellular level, and may help to optimize the timing of various therapeutic approaches. Cardiomyocytes isolated from adult Sprague-Dawley rats were exposed to Dox (10 μM), Vit C (25 μM), and Dox + Vit C for different time intervals up to 24 h. p38-JNK (SB203580) and p53 (pifithrin-α) inhibitors were used to determine the role of each respective signalling protein. Dox administration to cardiomyocytes increased the levels of ROS in a time-dependent manner that followed the activation of stress-induced proteins p53, p38 and JNK MAPKs, culminating in an increase in autophagy and apoptosis markers. Dox-induced increase in ROS was alleviated by Vit C adjuvant treatment at all time-points and this was also correlated with blunting of the activation of the studied signaling pathways leading to the prevention of apoptosis and preservation of cell viability. Protective effect of Vit C against the activation of stress induced proteins, autophagy and apoptosis was mainly attributed to its antioxidant properties even though blockage of p38, JNK and p53 by pharmacological inhibitors also suppressed Dox-induced apoptosis. ROS is defined as a key inducer of cardiomyocyte damage under Dox exposure; Vit C could effectively counteract all Dox-induced changes in cardiomyocytes and may potentially be used as an antioxidant adjuvant therapy to protect against Dox-induced cardiomyopathy.

## Introduction

The two leading causes of death worldwide, cardiovascular diseases and cancer [1,2], can be closely related considering that cancer survivors are at higher risk to develop heart failure due to the type of chemotherapy used [3]. The use of anticancer drug Doxorubicin (Dox) in cancer patients has been associated with a significant increase in the number of long-term cancer survivors [4]. For this reason, Dox is present in most chemotherapeutic cocktails, but its use is hampered by the serious dose-dependent side-effect of cardiotoxicity [5,6]. Thus, the life threatening Dox-induced cardiomyopathy is an important clinical problem. Unfortunately, no satisfactory clinically applicable preventive treatment is presently available. Therefore, the search for cardioprotective agents relies on the understanding of the molecular mechanisms involved and how to counteract them.

Dissociation of antitumor activity from the cardiotoxic effects of the drug has been documented [7,8]; which brings hope for the development of cardioprotective drugs that do not interfere with the antitumor activity. Several mechanisms underlying Dox-induced cardiotoxicity have been described; however, an increase in cardiac oxidative stress mediating the initial cardiac injury has gained support as the major cause of Dox-induced cardiomyopathy [8,9]. The heart’s unique vulnerability to oxidative stress after Dox treatment has been the focus of extensive amount of research [6, 10, 11, 12].

The activation of a number of focal signaling pathways has been identified as critical transducers of the cellular responses to stress such as oxidative stress to determine cell fate [13]. Amongst these regulators, the mitogen-activated protein kinases (MAPK) and tumor suppressor p53 have been linked to the anthracycline-induced response to the cardiac injury [14,15,16]. We have previously shown that Dox-induced injury in cardiomyocytes is linked to the generation of high levels of ROS, activation of MAPK p38 and tumor suppressor p53 [17,18]. However, their specific role and patterns of activation in Dox-induced cardiotoxicity needs further investigation in order to elucidate whether they can be targeted as candidates for therapeutic interventions. In addition, information on the beneficial effects of non-enzymatic antioxidant therapy to mitigate cell injury and apoptosis via attenuating over production of ROS and down-regulating stress induced proteins in adult cardiomyocytes exposed to Dox will address the need of a more specific adjuvant therapy.

Thus, the current study in rat cardiomyocytes investigated: i) the time-dependent generation of ROS, activation of p38, JNK and p53 leading to autophagy, apoptosis and cell death after Dox and Vit C treatments; and ii) the role of p38 and p53 inhibitors on Dox-induced changes. In some experiments, N-acetylcysteine (NAC) was used as positive control.

## Materials and methods

This study was approved by the University of Manitoba animal care and use committee following the guidelines established by the Canadian Council on Animal Care.

### Cardiomyocyte isolation and treatments

Cardiomyocytes were isolated from adult male Sprague—Dawley rats (250–300 g) using a previously described procedure (Lou et al. 2006) [19]. The hearts were excised and mounted on a modified Langendorff perfusion apparatus. The mounted hearts were perfused with calcium-free buffer (110 mM NaCl, 2.6 mM KCl, 1.2 mM KH_2_PO_4_, 1.2 mM MgSO_4_, 25 mM NaHCO_3_, 60 mM taurine, 11 mM glucose, pH 7.4) at 37°C to wash out the blood. Then the perfusion system was switched to the same buffer containing 0.1% collagenase, 0.1% BSA, 50 μmol CaCl_2_. Following the perfusion, ventricles were removed and incubated in desegregation solution containing 1% BSA and 50 μmol CaCl_2_. The ventricles were cut into small pieces and gently passed through pipettes with increasing concentration of CaCl_2_. The suspension was filtered through a nylon mesh (200 μM in size) and was allowed to settle for 10 min. The supernatant was discarded and the cell pellet was resuspended in medium 199 containing 1.8 mM CaCl_2_. Cardiomyocytes (10^6^ per dish) were plated in laminin-coated (20 μg/ml) polystyrene tissue culture dishes. Plated cells were incubated in culture medium 199 (M199) supplemented with 10% fetal bovine serum and antibiotics (streptomycin/penicillin, 100 μg/mL) at 37°C under a 5% CO_2_ and 95% O_2_ air atmosphere. This culture medium does not support the growth as well as survival of fibroblasts. Two hours after plating, the culture medium was changed to remove unattached dead cells, and the viable cardiomyocytes were incubated overnight.

### Validation of homogenous cardiomyocyte population

The procedure for the isolation of cardiomyocytes has been standardized in our laboratory [19]. The media used for cardiomyocyte isolation is M199, which is not conducive for the growth as well as survival of fibroblasts. Additionally, the attachment of fibroblasts requires ascorbic acid in the medium which was not available in the culture plates. The media is changed after 2 hours to remove unattached dead cells and the culture plate is checked under microscope to confirm the cardiomyocyte phenotype. It should also be note that, we always used freshly isolated cardiomyocytes as primary cultures. All these efforts as well as processes ensure that we are dealing principally with a homogenous cardiomyocyte population.

After initial incubation of 24 h, 95% of cardiomyocytes were viable and these cells were treated with one of the following in serum free medium: Control, cardiomyocytes cultured in M199 only; Vit C (25 μM) (SigmAldrich, Oakville, Canada); Dox (10 μM) (Adriamycin^®^, Pfizer, New York, USA); and combination of Vit C + Dox for up to 24 h, Vit C was added to M199 1 h prior to Dox treatment. The concentrations for Dox and Vit C used in the present study were carefully selected based on our previous studies [17,18].

In the time-course studies, cardiomyocytes were collected 1, 3, 6, 12 and 24h after incubation with different treatments. For the studies on the role of signalling pathways, cardiomyocytes were incubated with p38 inhibitor SB203580 (25 μM) (Sigma) or p53 inhibitor pifithrin-α (25 μM) (Sigma) with or without Dox (10 μM) for 24h. Inhibitors were added to M199 1 h prior to Dox treatment. Additionally, in some studies, cardiomyocytes were incubated with N-Acetyl Cysteine (NAC) (Fisher Scientific, Waltham, MA, USA) (50 μM), a water soluble antioxidant used as a positive antioxidant control. NAC concentration was defined based on pilot studies, and the concentration for the inhibitors was selected based on pilot studies and previously published work [20].

### Assessment of cardiomyocyte viability

Viability of the cultured cardiomyocytes was determined by staining cardiomyocytes after different treatments in culture dishes for 5 min at 37°C with trypan blue solution (Sigma) diluted to a final concentration of 0.04% (w/v). When cell membranes are irreversibly damaged, trypan blue, an anionic dye, is taken up by dead cells and binds to nuclei [21]. The cells were counted to quantify the percentage of rod shaped viable cells that did not stain blue.

### Measurement of ROS

The level of oxidative stress was monitored by the measurement of ROS. Cardiomyocytes from different treatment groups in the culture dishes were washed with phosphate-buffered saline (PBS) and incubated with 10 μM solution of fluorescent probe, 5-(6)-chloromethyl-dihydroflourescein diacetate (DCFDA) (Molecular Probes, Eugene, Oregon, USA) at 37°C for 30 min in a humidified chamber [17]. Fluorescent images from multiple fields per dish were recorded with the Olympus BX 51 fluorescent microscope. Fluorescence intensity was measured using digital image-processing software (Image Pro Plus). ROS production in the treated groups was represented as percentage of the mean optical density per unit area of the control group, defined as 100%.

### Western blot analysis

Whole-cell protein extracts were prepared from control and treated cardiomyocytes cultured in 60mm dishes. After different treatments, cells were washed in PBS and then placed on ice and RIPA lysis buffer (pH 7.6) containing phosphatase and protease inhibitors to lyse the cells and extract protein. The lysate was homogenized using a Polytron homogenizer and protein fraction was stored at ‾80°C.

The quantity of protein in the samples was measured by a dye-binding assay (Bio-Rad Laboratories, Hercules, CA, USA). The protein samples (45 μg) were then subjected to 12% SDS—PAGE and transferred to PVDF (Roche Diagnostics, Mississauga, ON, Canada) membrane [22]. Detection of PVDF membrane-bound proteins was performed using specific primary antibodies (Cell Signaling Technology inc., Mississauga, ON, Canada) for the study of signalling pathways (phospho-SAPK/JNK (Thr183/Tyr185), total SAPK/JNK, phospho-p38 MAPK (Thr180/Tyr182), total p38 MAPK, phospho p53 (ser 15), and total p53, as well as autophagy (LC3A/B antibody) and apoptotic markers (Bax, caspase-3 (8G10), PARP, Bcl-xL). Primary antibodies were detected using a goat anti-rabbit IgG horseradish peroxidase conjugate secondary antibody (Bio-Rad, Hercules, CA, USA). The membrane-bound proteins were visualized using a chemiluminiscence western blotting system (Roche Diagnostics) and developed on X-ray film. The bands were quantified by image analysis software (Quantity One, Bio-Rad, Hercules, CA, USA). Rabbit GAPDH antibody (Cell Signaling inc., Mississauga, ON, Canada) was used as loading control.

### Statistical analysis

All experiments were done in duplicates for each treatment group and time point, and repeated five times (n = 5). For the study of ROS, a total of 10 dishes (35 mm x 10 mm) were prepared for each treatment group and 10 different fields per dish were counted for the quantitative analysis. Data are expressed as the mean ± SEM. Groups were compared by one-way analysis-of-variance (ANOVA) or two-way ANOVA when more than one variable was analyzed for different groups, and the *post-hoc* Student-Newman-Keuls test was performed to identify differences between groups. P value of ≤ 0.05 was considered significant.

## Results

### Time-course effects of Dox and Vit C in cardiomyocytes

It is important to study the time-course changes due to Dox to understand the temporal changes leading to cardiomyocyte death and heart failure. For the time-course studies, cardiomyocytes were incubated with Dox for 5 different time durations (1, 3, 6, 12 and 24 h) with and without Vit C pre-treatment. At all time points, cell viability, ROS levels, stress-induced pathways and apoptotic markers were analyzed. In our pilot experiments, cardiomyocyte viability was assessed after 48h of Dox treatment (data not shown), however the number of viable cells obtained after this exposure time was below 50%. Furthermore, the reliability of data obtained in cultured adult cardiomyocytes is highly dependent on preservation of the original phenotype which can change after a prolonged culture as well as exposure time [23]. For these reasons, the 24 h mark was selected as the upper-limit time point in the time-course results.

### Cell viability

**Fig 1:**
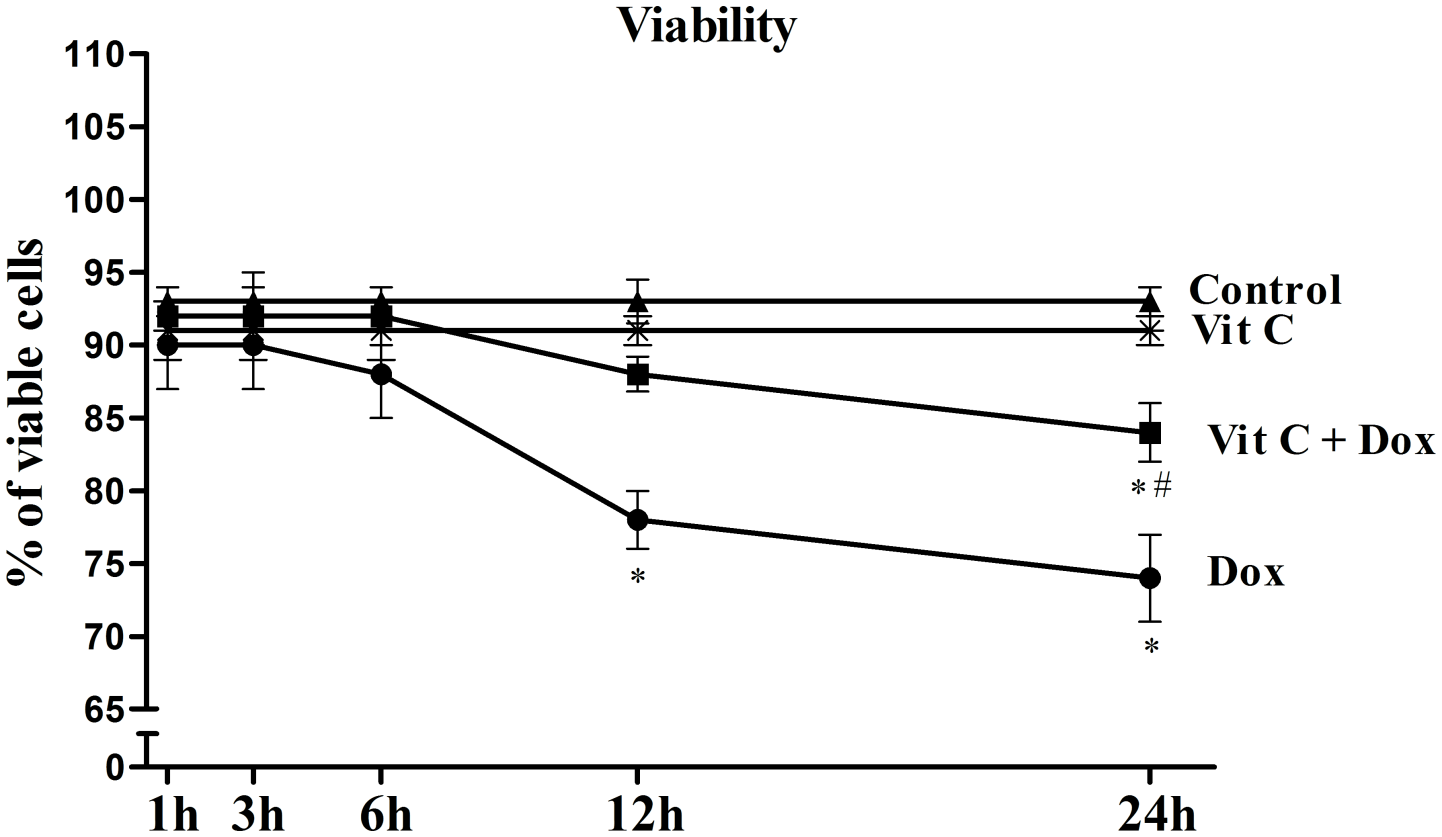
Dox-induced changes in cardiomyocyte viability with and without Vit C. Vit C (25 μM) was added 1 h prior to the addition of Dox (10 μM) for up to 24h. The data represented as percentage (%) of viable cardiomyocytes at different time points as the Mean±SEM of five different experiments done in duplicate. *Significantly different (P ≤ 0.05) from Control and Vit C and # significantly different (P ≤ 0.05) from the Dox group at the same time point.

In order to study the effect of Dox exposure at different time intervals, cell viability was measured after different time intervals. Dox treatment decreased the viability of cardiomyocytes and this change was significant (P≤0.05) at 12 and 24h of treatment (Fig 1). Pre-treatment with Vit C significantly reduced the Dox-induced loss of cell viability. With Vit C treatment, there was no cell loss due to Dox at 12h and the cell loss was significantly blunted at 24h (Fig 1. Dox-induced changes in cardiomyocyte viability with and without Vit C).

### ROS generation

**Fig 2:**
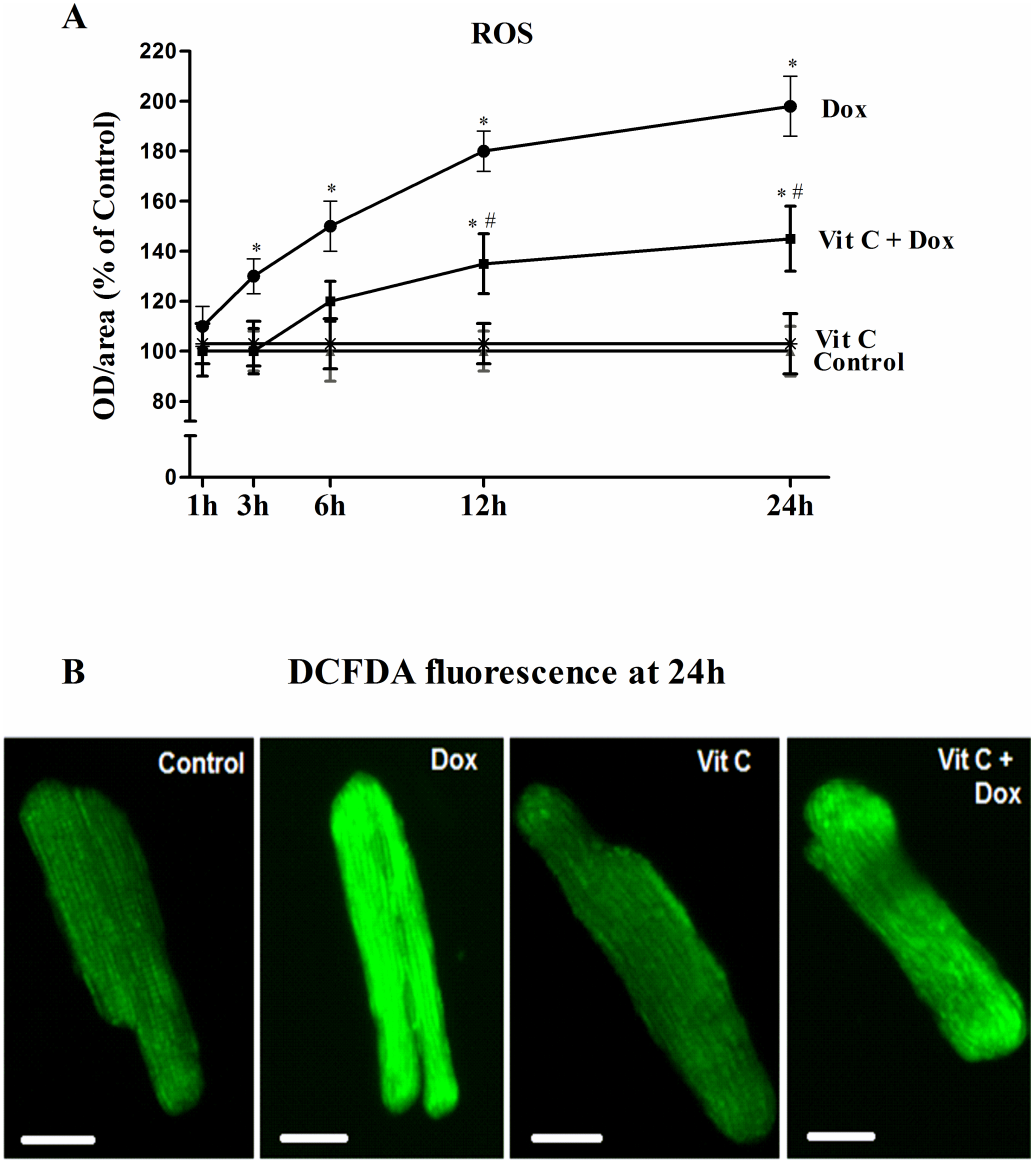
Levels of Dox-induced reactive oxygen species (ROS) and effects of Vit C (25 μM) as shown by DCFDA fluorescence. **A**) Changes in fluorescence intensity (% of Control) in cardiomyocytes at different time points; and **B**) representative images of cardiomyocytes at 24 h. Data are expressed as Mean± SEM of five different experiments done in duplicate. Scale bar represents 25 microns. *Significantly different (P≤ 0.05) from control and Vit C and # significantly different (P≤ 0.05) from the Dox group at the same time point.

Dox induced ROS generation is considered as one of the leading cause of cell death in the heart and subsequent heart failure. In our study, ROS levels were determined by the intensity of green fluorescent dye (DCFDA) in adult cardiomyocytes after different treatments. Dox treatment increased ROS levels in a time-dependent manner, and these levels were significantly higher (P≤0.05) than control as early as 3h in culture (Fig 2A and 2B). ROS production after Dox exposure progressively and significantly increased with time. Vit C was able to delay as well as blunt the sharp increase in ROS levels induced by Dox (Fig 2A). Representative images of cardiomyocytes stained with DCFDA after 24h of different treatments are shown in Fig 2B. There was 98±10.6% increase in green fluorescence at 24 h with Dox which was significantly reduced by pre-treatment with Vit C (Fig 2. Levels of Dox-induced Reactive Oxygen Species (ROS) and the effects of Vit C as shown by DCFDA fluorescence).

### Stress-induced pathways

**Fig 3:**
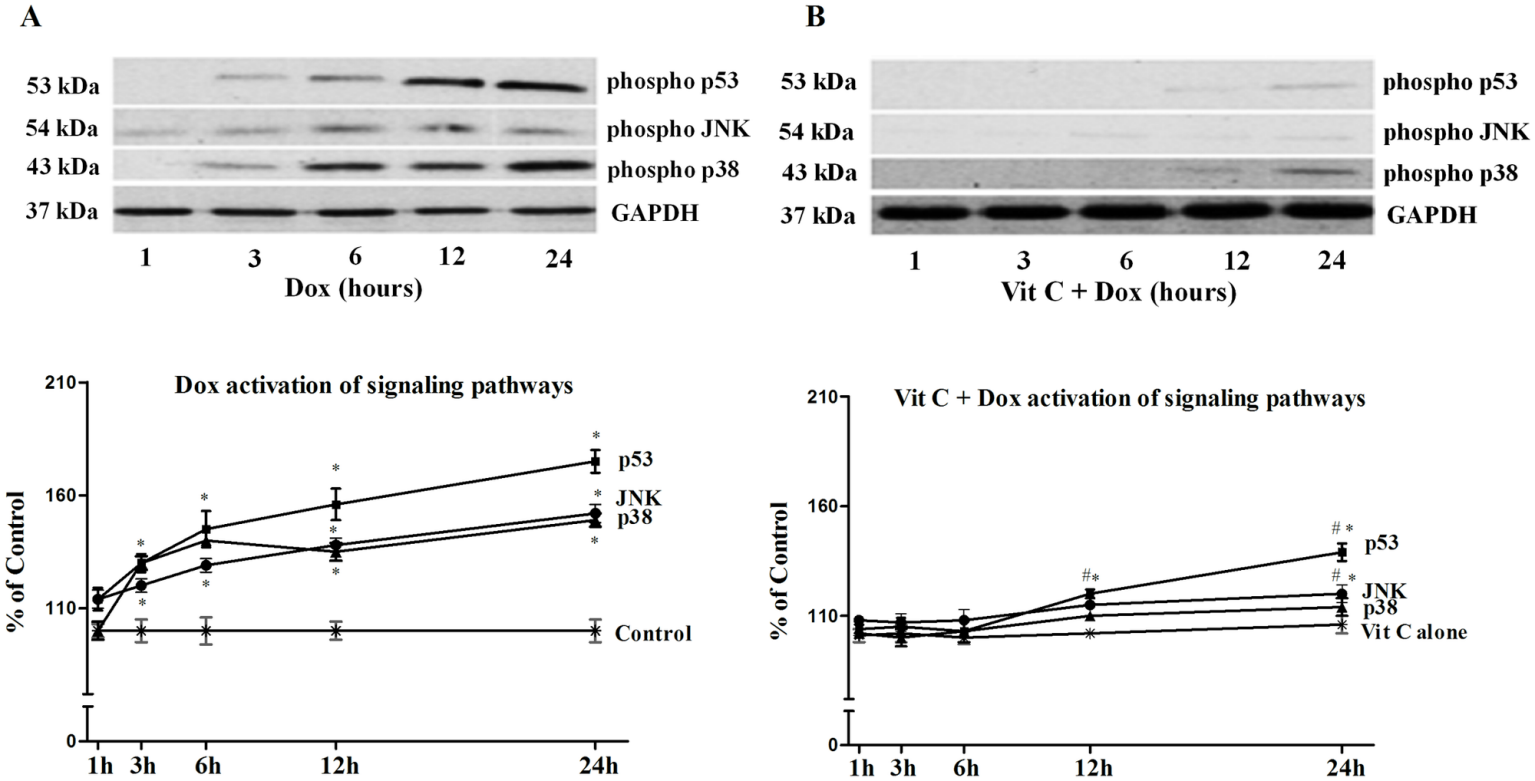
Effects of Vit C on Dox-induced changes in stress-induced proteins (p53, JNK and p38 MAPK) in cardiomyocytes at different time intervals. **A**) Changes due to Dox alone; and B) Changes in Vit C + Dox group. Upper panels are westen blot representations of phosphorylated proteins and lower panels are densitometric analysis of these phosphorylated proteins. Activation of the analyzed signaling pathways was compared against control, set as 100%. Data are expressed as mean±SEM of five different experiments done in duplicate. *Significantly different (P < 0.05) from Control or Vit C and # significantly different (P < 0.05) from the Dox group at the same time point.

Activation of stress-induced pathways such as mitogen-activated protein kinases (MAPK) and tumor suppressor p53 is identified as critical transducers of the cellular responses to stress such as oxidative stress, cell injury and death. The time course effects of Dox with and without Vit C were studied for up to 24h for different stress-induced proteins (p53, JNK and p38) and these data are shown in Fig 3A and 3B. After Dox treatment, JNK phosphorylation was detected within the first hour of Dox exposure and this activation was gradually increased from 6 to 24 h; p38 and p53 phosphorylation became evident within the first 3 hours, being significantly more pronounced at 6 to 24h (Fig 3A). These Dox-induced patterns were significantly changed when cardiomyocytes were pre-treated for 1h with Vit C (Fig 3B) where the activation of the proteins p38, JNK and p53 due to Dox was significantly blunted by Vit C adjuvant treatment. Control values were set as 100% and no differences were seen in control and Vit C groups (Fig 3. Effect of Vit C on Dox-induced changes in stress-induced proteins (p53, JNK and p38 MAPK) in cardiomyocytes at different time intervals).

### Apoptotic markers

**Fig 4:**
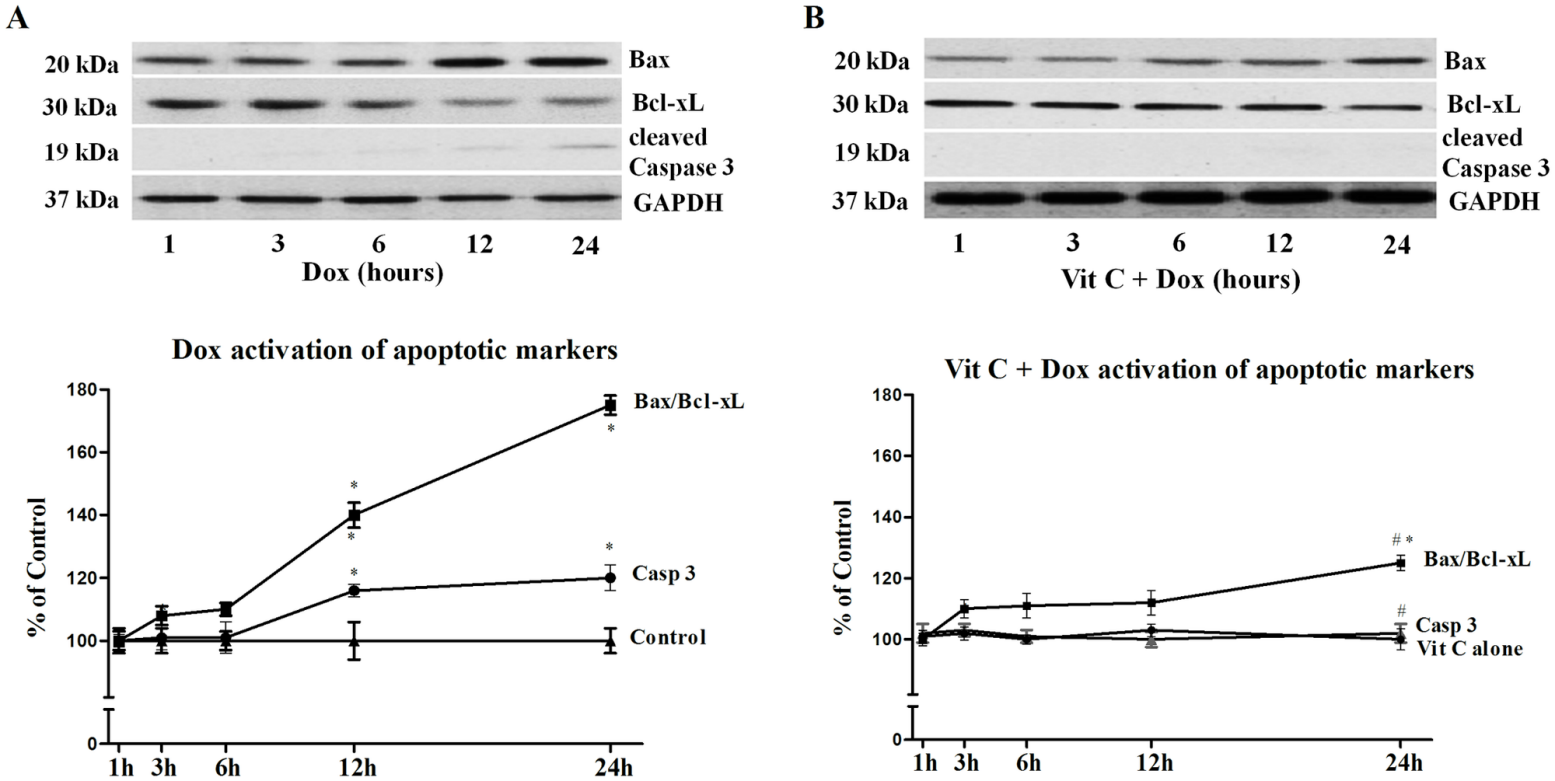
Time-course effects of Vit C on Dox-induced changes in apoptotic markers (Bax/Bcl-xl ratio and Caspase-3 cleavage) in cardiomyocytes. **A**) changes due to Dox alone; and **B**) changes in Vit C + Dox group. Upper panels are westen blot representations of proteins and lower panels are densitometric analysis of these proteins. Activation of the analyzed proteins was compared against Control, set as 100%. Data are expressed as mean±SEM of five different experiments. *Significantly different (P<0.05) from Control or Vit C and # significantly different (P<0.05) from the Dox group at the same time point.

Since apoptosis is one of the crucial detrimental effects of Dox in the heart, the time-course effects (1 to 24h) of Dox and the influence of Vit C on apoptotic markers were studied in isolated cardiomyocytes using western blot where the ratio of pro-apoptotic protein Bax over the anti-apoptotic protein Bcl-xL was quantified along with cleaved caspase-3, an indicator of caspase-3 activation (Fig 4A and 4B). There was a time-dependent rise in the pro-apoptotic protein Bax and decline in anti-apoptotic protein Bcl-xL (Fig 4A). At 12 and 24h of Dox exposure, the increase in Bax expression and decrease in Bcl-xL expression resulting in an increase of the Bax/Bcl-xL ratio was significant (P≤0.05) as compared with the control (Fig 4A). Vit C treatment significantly (P≤ 0.05) inhibited this Dox-induced rise in Bax/Bcl-xL ratio (Fig 4B). Cleavage of caspase-3 due to Dox was noticed after 12 and 24h in culture (Fig 4A). In the presence of Vit C, caspase-3 activation was completely abolished in Dox treated cardiomyocytes (Fig 4B). Control values were set as 100% and no differences were seen in control and Vit C groups (Fig 4. Time course effects of Vit C on Dox-induced changes in apoptotic markers (Bax/Bcl-xl ratio and Caspase 3 cleavage) in cardiomyocytes).

### Antioxidant effect of Vit C on Dox-induced changes in stress induced pathways, apoptosis and autophagy

**Fig 5:**
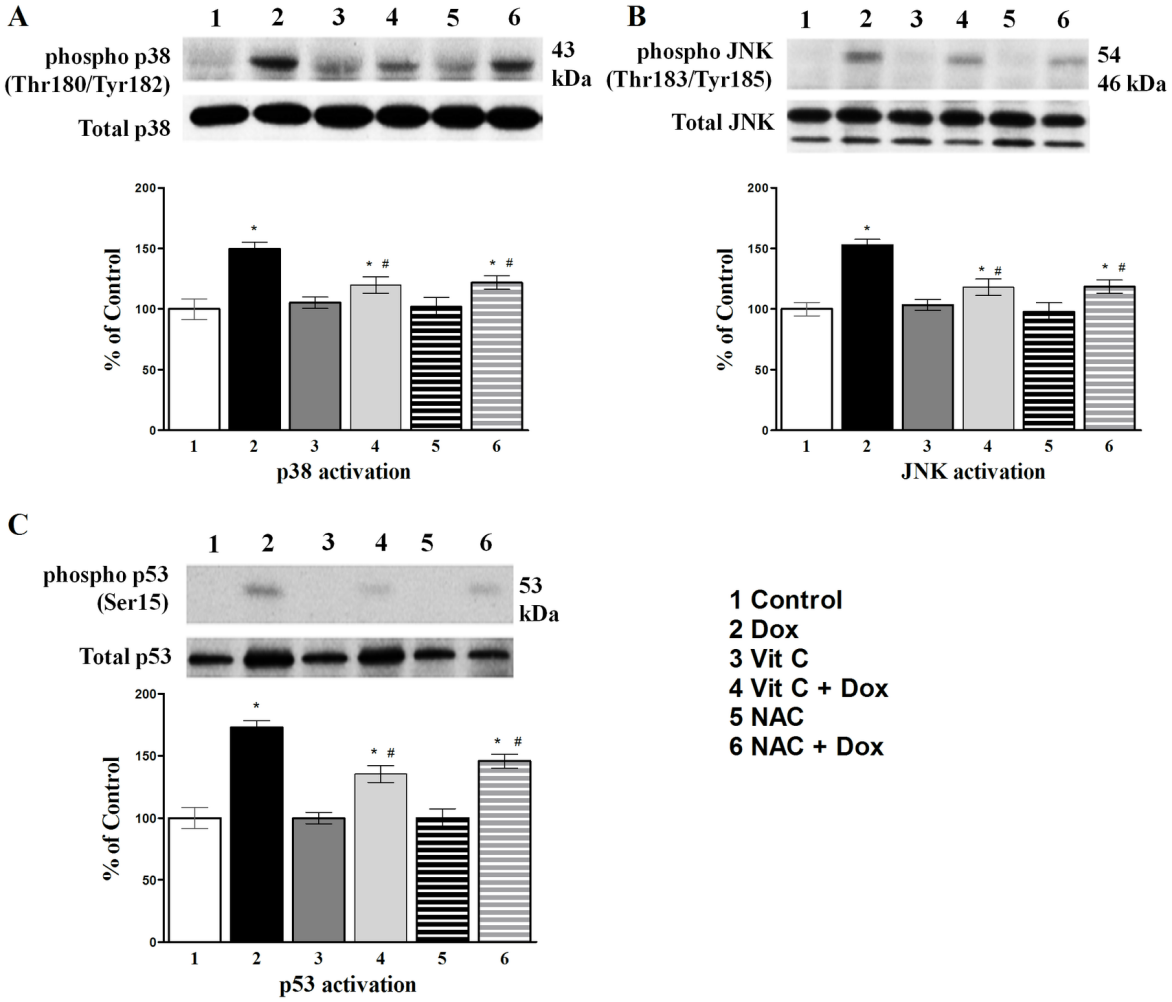
Effects of different treatments on stress-induced signaling pathways p38 (A), JNK (B), and p53 (C) in cardiomyocytes. In all figures, upper part is the Western blot analysis and the lower part shows densitometric analysis (% of control). Vit C (25 μM) or NAC (50 μM) were added 1 hour prior to the addition of Dox (10 μM) for 24h. Data are expressed as Mean±SEM of five different experiments done in duplicates. *Significantly different (P<0.05) from control and # significantly different (P< 0.05) from the Dox group.

In order to understand whether Vit C mediated protection against Dox-induced changes in stress-related signaling pathways, autophagy and apoptosis could be attributed mostly to its antioxidant properties, a well-known water soluble antioxidant, N-acetylcyteine (NAC), was used as positive control at the selected working dose of 50 μM and the duration for Dox exposure was 24h. Vit C and NAC were added to the culture media 1h prior to the addition of Dox. The activation of p38 and JNK MAPKs as well as p53 was expressed as the ratio of the phosphorylated to total protein (Fig 5A–5C). Dox-induced p38, JNK and p53 activations were significantly reduced in the presence of antioxidants, Vit C or NAC (Fig 5. Effects of different treatments on stress-induced signaling pathways p38 (**A**), JNK (**B**), and p53 (**C**) in cardiomyocytes).

**Fig 6:**
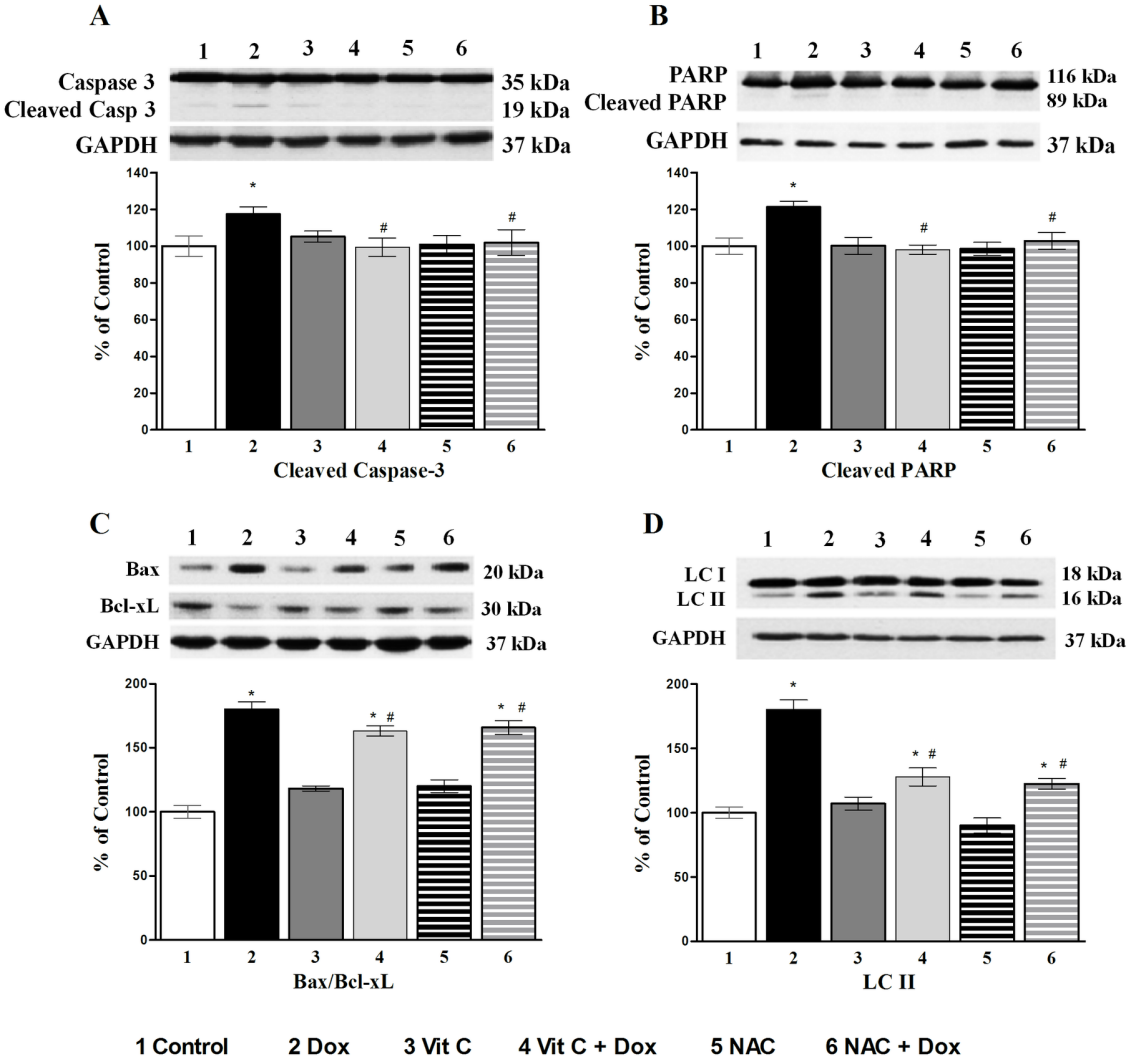
The effects of different treatments for 24 h on apoptotic markers. A) caspase-3 cleavage; B) PARP cleavage; C) Bax/Bcl-xL ratio; and **D**) autophagic marker, LC3 II in cardiomyocytes. In all figures, upper part is the western blot analysis and the lower part shows densitometric analysis (% of control). Data are expressed as mean±SEM of five different experiments done in duplicate. *Significantly different (P<0.05) from control and # significantly different (P<0.05) from the Dox group.

In order to investigate apoptosis and autophagy, further evidence for the confirmation of caspase-3 activation was obtained by monitoring the cleavage of its substrate, PARP along with Bax/Bcl-xL ratio. Dox-induced cleavage of caspase-3 and PARP was abolished by antioxidant treatments for 24 h (Fig 6A and 6B). The significant increase in Bax/Bcl-xL ratio caused by Dox was alleviated by Vit C or NAC when compared to control (Fig 6C). When autophagy is induced, microtubule-associated protein, light chain 3 (LC3), is processed from LC3-I (18 kDa) to LC3-II (16 kDa) and incorporated into autophagic vacuoles. The Western blot analysis showed an increase in the formation of LC3-II after Dox treatment (Fig 6D), suggesting that autophagy activation is supporting apoptosis, and this change was blunted by pre-treatment with Vit C or NAC. Control values were set as 100% and no differences were seen in control and Vit C groups. (Fig 6. The effects of different treatments for 24 h on apoptotic markers: A) Caspase 3 cleavage; B) PARP cleavage; C) Bax/Bcl-xL ratio; and D) autophagic marker, LC3 II in cardiomyocytes).

### Cell viability and the generation of ROS in the presence of MAPK and p53 inhibitors

In order to understand the importance of MAPKs and p53 activation, cells were cultured with SB203580 (pharmacological inhibitor of p38 that also inhibits JNK in the concentration used), and pifitrin-α (pharmacological inhibitor of p53) 1h prior to addition of Dox for 24h. Treatment with p38/JNK and p53 inhibitors abrogated Dox-induced decrease in cardiomyocyte viability and blunted the Dox-induced increase in the ROS production (Table 1).

**Table 1:** Viability and ROS production in cardiomyocytes treated with doxorubicin with and without inhibitors of different signalling pathway inhibitors.

Groups	Viability	ROS production (% control)
(i) Control	91.5 ± 3.2	100
(ii) Dox (10μM)	71 ± 2.1*	198 ± 10.6*
(iii) p38 inhibitor (25μM)	90.2 ± 3.1	105 ± 8.9
(iv) Dox (10μM) + p38 inhibitor (25μM)	80 ± 3.5*^#^	175 ± 7.5*^#^
(v) p53 inhibitor (25μM)	90.8 ± 3.5	103 ± 9.8
(vi) Dox (10μM) + p53 inhibitor (25μM)	81.2 ± 3.1*^#^	170 ± 8.8*^#^

ROS levels were analyzed by DCFDA fluorescence intensity and expressed as percentage of control. Cell viability was quantified using trypan blue dye to differentiate viable and dead cells (percentage of viability). Cardiomyocytes were cultured for 24h: (i) Control- cardiomyocytes cultured in M199; (ii) Dox- 10 μM of doxorubicin; (iii) p38 inhibitor with 25 μM of SB203580; (iv) Dox (10 μM) + p38 inhibitor (25 μM); (v) p53 inhibitor with 25 μM of pifithrin-α; and (vi) Dox (10 μM) + p53 inhibitor (25 μM).

Data are expressed as Mean±SEM of five different experiments done in duplicates.

*Significantly different (P<0.05) from Control and

^#^ significantly different (P<0.05) from the Dox group.

### Effects of MAPK and p53 inhibition on apoptotic markers

**Fig 7:**
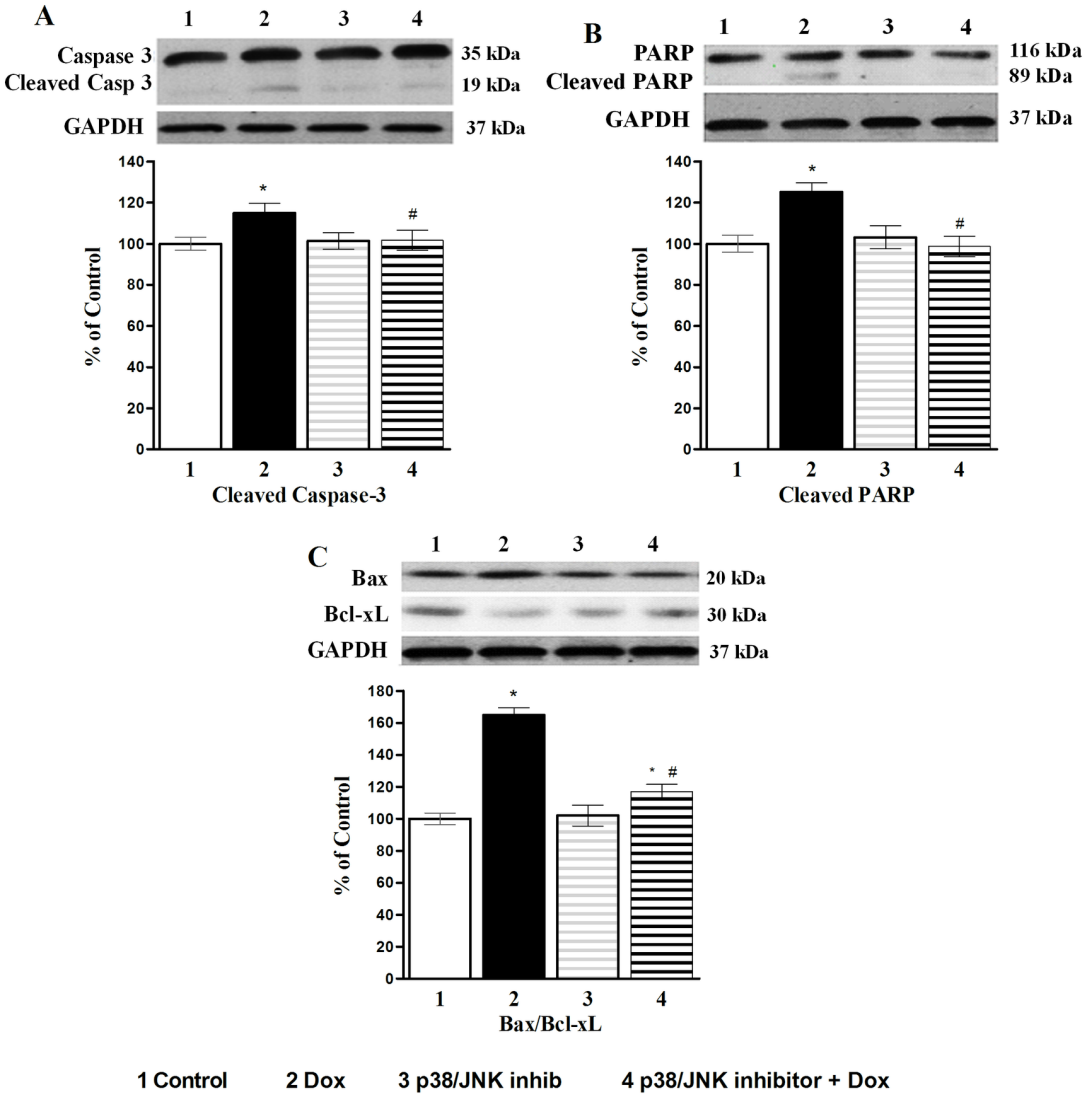
Effects of p38/JNK inhibition on apoptotic markers. **A**) Caspase 3 cleavage; **B**) PARP cleavage and **C**) Bax/Bcl-xL ratio in cardiomyocytes. In all figures, upper part is the Western blot analysis and the lower part shows densitometric analysis (percentage of control). SB203580 (25 μM), p38 inhibitor was added 1 hour prior to the addition of Dox (10 μM) for 24h. Data are expressed as Mean±SEM of five different experiments done in duplicate. *Significantly different (P<0.05) from control and # significantly different (P<0.05) from the Dox group.

We further investigated the role of p38/JNK inhibition on specific parameters of apoptosis. Inhibition of p38/JNK with SB203580 was able to prevent Dox-induced cleavage of caspase-3 and PARP as well as blunted the increase in the pro-apoptosis index Bax/Bcl-xL ratio (Fig 7. Effects of p38/JNK inhibition on apoptotic markers: A) Caspase 3 cleavage; B) PARP cleavage and C) Bax/Bcl-xL ratio in cardiomyocytes).

**Fig 8:**
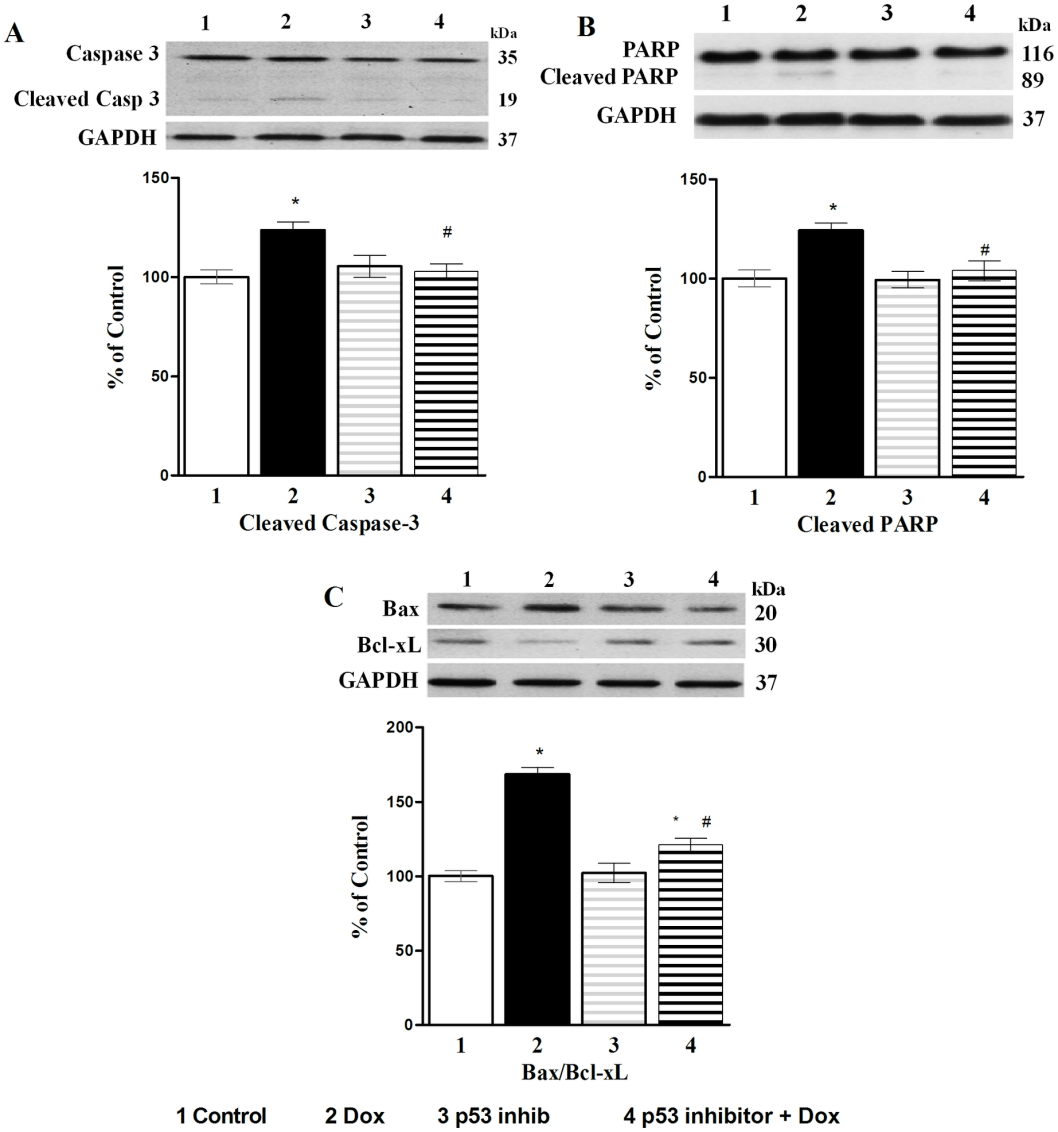
Effects of p53 inhibition on apoptotic markers. **A**) Caspase-3 cleavage; **B**) PARP cleavage; and **C**) Bax/Bcl-xL ratio in cardiomyocytes. In all figures, upper part is the Western blot analysis and the lower part shows densitometric analysis (% control). pifithrin-α (25 μM), p53 inhibitor was added 1 hour prior to the addition of Dox (10 μM) for 24h. Data are expressed as Mean±SEM of five different experiments done in duplicate. *Significantly different (P≤0.05) from Control and # significantly different (P<0.05) from the Dox group.

Effects of p53 inhibition on apoptosis due to Dox are shown in Fig 8A–8C. Dox-induced cleaved caspase-3 and PARP activities as well as Bax/Bcl-xL ratio were decreased by pifitrin-α indicating that Dox-induced apoptosis is also influenced by p53 activation. Control values were set as 100% and no differences were seen in control and Vit C groups (Fig 8. The effects of p53 inhibition on apoptotic markers Caspase-3 cleavage (A), PARP cleavage (B), and Bax/Bcl-xL ratio (C) in cardiomyocytes).

## Discussion

Dox-induced cardiomyopathy still remains an important clinical problem [3,4]. Research on the underlying mechanisms of this unique cardiomyopathy is crucial to develop therapeutic strategies and prevent premature cardiac cell loss in patients subjected to anthracycline treatment. In order to understand the temporal changes leading to cardiomyocyte loss, we studied the time-course changes due to Dox by evaluating oxidative stress, stress-induced pathways and apoptosis. Furthermore, time dependant studies on signalling proteins help to identify the pathophysiology as well as regulation of Dox-mediated cardiomyopathy at the cellular level and will help to optimize the timing of different therapeutic approaches. Antioxidant Vit C was used as a cardioprotective strategy and NAC was used as a positive control. The role of p38/JNK and p53 was also addressed using specific pharmacological inhibitors to understand whether these proteins could be used as targeted therapy against Dox-induced cardiotoxicity.

The concentration of 10 μM of Dox used in the present study has been reported to be effective in its antitumor activity as well as effective in a cumulative dose [24]. At this dose of Dox, our time-course studies demonstrated an increase in ROS, followed by activation of the signaling proteins such as MAPKs and p53. This was later associated with an increase in apoptotic markers shown by an increase in Bax/Bcl-xL ratio and caspase-3 activation, impairing cardiomyocyte viability. It is interesting to note that cardiomyocyte viability was significantly reduced at 12 and 24 hours after Dox treatment when ROS production was also significantly elevated above the 50% mark from control. Thus, a high formation of ROS precedes the apoptotic cell death.

Our findings also show that the phosphorylation of p38 and JNK MAPKs presented a gradual and persistent increase for up to 24 h. These findings are supported by pre-clinical studies where it has been reported that these MAPKs increase early and persist until the heart failure stage, suggesting that these pathways may play an important role in the progression of anthracycline-induced cardiomyopathy and heart failure [15,19]. In agreement with our data, other studies have also shown that Dox-induced oxidative stress activates various signaling pathways which culminate in apoptotic cell death contributing to cardiomyopathy [25,26]. In this regard, it has already been suggested that ROS activates MAPK family proteins [15,24]. Among them, p38 and JNK MAPKs may play critical roles in Dox-induced cell death pathways [25]. Depending on the time duration, types of stress and stimuli, MAPKs respond rapidly or permanently in the heart, to mediate cellular damage and cardiac dysfunction in response to oxidative stress. Even though the pharmacological inhibition of p38/JNK MAPks also blunted the increase in ROS levels, stress-induced pathways p38 and JNK MAPKs were shown to be activated after Dox-induced increase in ROS levels, and they may participate in a positive feedback leading to more ROS production and enhancement of aoptotic cell death. Thus their inhibition can lead to less oxidative stress due to blockade of this positive feedback. In the present study, pharmacological inhibition of p38/JNK MAPKs abolished the increase in apoptotic markers Bax/Bcl-xL ratio, caspase-3 and PARP cleavage supporting the role of these kinases in Dox-induced apoptosis. Both p38 MAPK and JNK have been shown to induce Bax activation in human hepatoma HepG2 and porcine kidney LLC-PK1 cells exposed to various cell death agonists [27] and in cardiomyocytes exposed to Dox [25].

Thus, considerable data indicate that cardiomyocyte death through apoptosis and necrosis occurs after Dox exposure; however other forms of cellular dysfunction have emerged and autophagy is postulated to be an important contributor to the progression of Dox-induced cardiomyopathy [28]. In fact, an increase in the autophagic marker LC3 II, which is currently used as an indicator of the number of autophagosomes [29] after Dox exposure was seen to rise in the present study indicating autophagy might have a role in the cell death process due to Dox. In this regard, dysregulation of autophagy in the myocardium was also recently reported in Dox-induced cardiotoxicity [30]. Taken together, these results reiterate that cardiomyocyte dysfunction after Dox treatment is complex and may result from the activation of various pathways that include oxidative/nitrosative stress, mitochondrial damage, DNA damage, and induction of pro-apoptotic proteins and cell membrane injury [31,32].

One of the key regulator of cellular dysfunction and stress is tumour suppressor protein p53 which can either induce cell survival or death depending on the nature of cells and type of stress involved [25,33]. In Dox-induced cardiotoxicity, high doses of Dox were shown to induce p53 activation in cardiomyocytes which was closely associated with apoptosis [34,35]. In the present study, phosphorylation of p53 started to increase at 3 hours after Dox treatment, subsequent to the increase seen in ROS. A previous study performed in neonatal rat cardiomyocytes exposed to 1 μM of Dox suggested that p53 activation is via oxidative DNA damage and it was also associated with apoptosis. In their study, pitavastatin attenuated Dox-induced cardiotoxicity through antioxidant effects [35]. In the present study, treatment with antioxidant Vit C and pifitrin-α, the pharmacological inhibitor of p53, abrogated Dox-mediated decrease in cell viability and increase in apoptotic markers in cardiomyocytes. These findings indicate that Dox is an inducer of p53 activation, and stimulates cardiomyocyte death in a p53-dependent manner.

It is well known that the quinone moiety of Dox can be reduced by oxido-reductases producing a semiquinone radical associated with a redox cycling leading to reactive forms of oxygen [12]. ROS mediated damage largely occurs via lipid peroxidation as well as reduction in thiol groups [3,7]. Lipid peroxidation results in changes in membrane architecture and permeability as well as alterations in membrane bound enzyme activities. Every compartment of the cell surrounded by membranes can be affected. Vit C is considered to be one of the most potent and least toxic antioxidants for humans [36]. Vit C has the ability to scavenge reactive oxygen, nitrogen and chlorine species, thereby effectively protecting cellular substrates from oxidative damage. Although earlier studies on animals have shown that Vit C reduced cardiotoxicity and prolonged life after Dox exposure [37,38,39,40], there is little information about subcellular basis of this beneficial effect. The present study provides a better understanding of the molecular mechanisms of antioxidant, vit C against Dox-induced changes. Our data showed that the presence of Vit C prior to the addition of Dox to cardiomyocytes was able to minimize oxidative stress and prevent the decrease in cell viability due to the drug toxicity. Most importantly, Vit C delayed as well as reduced Dox-induced activation of stress-induced pathways, ROS production and the rise in apoptotic markers, partially rescuing cardiomyocyte loss. Our findings showed that Vit C was not only able to prevent apoptosis but also autophagy. As NAC, another water-soluble antioxidant also showed protection against Dox-induced changes in cardiomyocytes, it is likely that the beneficial effects of Vit C are due to its antioxidant properties. Thus the results from the present study clearly established the preventive effects of Vit C in attenuating Dox-induced cell injury in cardiomyocytes. Furthermore, cancer patients who generally present lower concentrations of Vit C in plasma [41] could have additional benefits from its supplementation. Herein, we provide a strong basis for the design of further animal and clinical studies on Vit C as adjuvant therapy in Dox-induced cardiomyopathy.

## Supporting information

S1 File Raw data and uncropped western blot images. **Table A)** Time dependent changes in the viability of Dox-treated cardiomyocytes with and without Vit C. **Table B)** Time dependent changes in the reactive oxygen species (ROS) generation in Dox-treated cardiomyocytes with and without Vit C. **Table C)** Time dependent changes in the levels of stress-induced proteins (p53, JNK and p38 MAPK) in Dox treated cardiomyocytes. **Table D)** Time-dependent changes in apoptotic markers (Bax/Bcl-xl ratio and Caspase-3 cleavage) in Dox treated cardiomyocytes with or without Vit C. **Table E)** Changes in the expression of stress-induced signaling proteins (p38, JNK and p53) in different treatment groups. **Table F)** Dox-dependent changes in the expression of apoptotic proteins in different treatment groups. **Table G)** Effects of p38 inhibition on apoptotic markers. **Table H)** Effects of p53 inhibition on apoptotic proteins PARP, Caspase-3 and Bax/Bcl-xl was determined in different treatment groups. Data are expressed as Mean±SEM of five different experiments done in duplicate. *Significantly different (P<0.05) from control and # significantly different (P<0.05) from the Dox group. **Figure A)** Representative uncropped western blot images. i) GAPDH; ii) phospho JNK; iii) total p38; iv) phospho p53; v) Bcl-xl and Bax; vi) Total and cleaved caspase-3; vii) LC3; viii) time course for Bcl-xl and Bax; ix) time course for phospho p 53; x) time course for phospho p38; xi) Bcl-xl with MAPK inhibitor; xii) Total and cleaved PARP withMAPK inhibitor; and xiii) Bax with MAPK inhibitor.
(DOCX)
