# Supplementary material for: Time course of changes in oxidative stress and stress-induced proteins in cardiomyocytes exposed to doxorubicin and prevention by vitamin C
Source: PLoS One. 2017 Jul 5;12(7):e0179452. doi: 10.1371/journal.pone.0179452 (PMC5497966; doi:10.1371/journal.pone.0179452)
Supplement: S1 File — Table A) Time dependent changes in the viability of Dox-treated cardiomyocytes with and without Vit C. Table B) Time dependent changes in the reactive oxygen species (ROS) generation in Dox-treated cardiomyocytes with and without Vit C. Table C) Time dependent changes in the levels of stress-induced proteins (p53, JNK and p38 MAPK) in Dox treated cardiomyocytes. Table D) Time-dependent changes in apoptotic markers (Bax/Bcl-xl ratio and Caspase-3 cleavage) in Dox treated cardiomyocytes with or without Vit C. Table E) Changes in the expression of stress-induced signaling proteins (p38, JNK and p53) in different treatment groups. Table F) Dox-dependent changes in the expression of apoptotic proteins in different treatment groups. Table G) Effects of p38 inhibition on apoptotic markers. Table H) Effects of p53 inhibition on apoptotic proteins PARP, Caspase-3 and Bax/Bcl-xl was determined in different treatment groups. Data are expressed as Mean±SEM of five different experiments done in duplicate. *Significantly different (P<0.05) from control and # significantly different (P<0.05) from the Dox group. Figure A) Representative uncropped western blot images. i) GAPDH; ii) phospho JNK; iii) total p38; iv) phospho p53; v) Bcl-xl and Bax; vi) Total and cleaved caspase-3; vii) LC3; viii) time course for Bcl-xl and Bax; ix) time course for phospho p 53; x) time course for phospho p38; xi) Bcl-xl with MAPK inhibitor; xii) Total and cleaved PARP withMAPK inhibitor; and xiii) Bax with MAPK inhibitor. (DOCX) [file pone.0179452.s001.docx]

**Table A:**

|  | **Groups** | **1h** | **3h** | **6h** | **12h** | **24h** |
| --- | --- | --- | --- | --- | --- | --- |
| **Viability** | Control | 93±1 | 93±2 | 93±1 | 93±1.5 | 93±1 |
|  | Dox | 90±8 | 90±7 | 88±10 | 80±8^*^ | 75±12^*^ |
|  | Vit C | 91±2 | 91±2 | 91±2 | 91±1 | 91±1 |
|  | Vit C + Dox | 92±0.09 | 92±1 | 92±2 | 88±1.2 | 88±2^*#^ |

**Table B:**

|  | **Groups** | **1h** | **3h** | **6h** | **12h** | **24h** |
| --- | --- | --- | --- | --- | --- | --- |
| **ROS** | Control | 100±10 | 100±8 | 100±12 | 100±8 | 100±10 |
|  | Dox | 110±8 | 130±7^*^ | 150±10^*^ | 180±8^*^ | 198±12^*^ |
|  | Vit C | 103±8 | 103±9 | 103±10 | 103±8 | 103±12 |
|  | Vit C + Dox | 100±10 | 100±9 | 120±8 | 135±12^*#^ | 145±13^*#^ |

**Table C:**

|  | **Groups** | **1h** | **3h** | **6h** | **12h** | **24h** |
| --- | --- | --- | --- | --- | --- | --- |
| **JNK** | Control | 100±3 | 100±5 | 100±6 | 100±4 | 100±5 |
|  | Dox | 114±4 | 120±3^*^ | 129±3^*^ | 138±3^*^ | 152±4^*^ |
|  | Vit C | 103±3 | 102±4 | 100±3 | 104±2 | 105±4 |
|  | Vit C + Dox | 108±2 | 107±4 | 108±5 | 115±2 | 120±4^*#^ |
|  |  |  |  |  |  |  |
| **p53** | Control | 100±8 | 100±4 | 100±5 | 100±6 | 100±8 |
|  | Dox | 114±5 | 130±4^*^ | 145±8^*^ | 156±7^*^ | 175±5^*^ |
|  | Vit C | 101±3 | 102±4 | 100±3 | 102±2 | 106±4 |
|  | Vit C + Dox | 104±2 | 105±4 | 103±5 | 120±2^*#^ | 139±4^*#^ |
|  |  |  |  |  |  |  |
| **p38** | Control | 100±4 | 100±6 | 100±7 | 100±5 | 100±4 |
|  | Dox | 100±4 | 130±3^*^ | 140±2^*^ | 135±4^*^ | 149±3^*^ |
|  | Vit C | 103±3 | 102±4 | 100±3 | 102±2.5 | 103±4 |
|  | Vit C + Dox | 102±2 | 100±4 | 103±5 | 110±2 | 114±4 |

**Table D:**

|  | **Groups** | **1h** | **3h** | **6h** | **12h** | **24h** |
| --- | --- | --- | --- | --- | --- | --- |
| **Caspase-3** | Control | 100±3 | 100±4 | 100±3 | 100±6 | 100±4 |
|  | Dox | 100±2 | 101±4 | 101±5 | 116±2^*^ | 120±4^*^ |
|  | Vit C | 102±3 | 103±2 | 101±2.1 | 100±2.5 | 102±3 |
|  | Vit C + Dox | 101±2 | 102±2.2 | 100±1.5 | 103±2 | 100±3.5^#^ |
|  |  |  |  |  |  |  |
| **Bax/Bcl-xl** | Control | 0.5±0.03 | 0.51±0.05 | 0.52±0.05 | 0.5±0.1 | 0.52±0.08 |
|  | Dox | 0.5±0.05 | 0.54±0.02 | 0.55±0.04 | 1.2±0.03^*^ | 1.3±0.05^*^ |
|  | Vit C | 0.54±0.01 | 0.55±0.008 | 0.5±0.008 | 0.55±0.01 | 0.55±0.08 |
|  | Vit C + Dox | 0.51±0.009 | 0.5±0.012 | 0.56± | 0.55±0.03 | 0.85±0.05^*#^ |

**Table E:**

| **Groups** | **JNK** | **p53** | **p38** |
| --- | --- | --- | --- |
| Control | 100±5.6 | 100±8.5 | 100±8.5 |
| Dox | 152.68±4.94^*^ | 172.79±5.6^*^ | 149.6±5.6^*^ |
| Vit C | 103±4.6 | 100±4.6 | 105.33±4.6 |
| Vit C + Dox | 108±6.8^*#^ | 135.44±6.8^*#^ | 120±6.8^*#^ |
| NAC | 97.75±7.5 | 100±7.5 | 101.98±7.5 |
| NAC + Dox | 118.46±5.6^*#^ | 145.87±5.6^*#^ | 121.88±5.6^*#^ |

**Table F:**

|  | **Groups** | **Cleaved PARP** | **Cleaved Caspase-3** | **Bax/Bcl-xl** | **LC-3** |
| --- | --- | --- | --- | --- | --- |
| **Cell death** | Control | 100±4.5 | 100±5.5 | 100±5 | 100±4.5 |
|  | Dox | 121.16±3.2^*^ | 117.32±4^*^ | 250.72±6 | 179.84±8^*^ |
|  | Vit C | 100.20±4.6 | 105.22±3 | 118.31±2 | 107.11±5.1 |
|  | Vit C + Dox | 98.14±2.5^#^ | 99.43±5^#^ | 163.59±4 | 128±7^*#^ |
|  | NAC | 98.57±3.5 | 100.87±4.8 | 128.13±5 | 90.11±6 |
|  | NAC + Dox | 102.80±4.5^#^ | 101.92±7^#^ | 198.38±8 | 122.62±4.1^*#^ |

**Table G:**

|  | **Groups** | **Cleaved PARP** | **Cleaved Caspase-3** | **Bax/Bcl-xl** |
| --- | --- | --- | --- | --- |
| **p38 inhibitor** | Control | 100±3.5 | 100±3.2 | 100±3.6 |
|  | Dox | 125.29±4.3^*^ | 115.32±4.6^*^ | 165±4.5^*^ |
|  | p38 inhibitor | 103.19±5.6 | 101.33±4 | 102±6.5 |
|  | P38 inhibitor + Dox | 98.64±5^#^ | 101.73±5^#^ | 117±4.5^*#^ |

**Table H:**

|  | **Groups** | **Cleaved PARP** | **Cleaved Caspase-3** | **Bax/Bcl-xl** |
| --- | --- | --- | --- | --- |
| **p53 inhibitor** | Control | 100±4.2 | 100±3.6 | 100±3.6 |
|  | Dox | 124.28±3.6^*^ | 123.51±4.2^*^ | 168±4.5^*^ |
|  | p53 inhibitor | 99.31±4.1 | 105.35±5.6 | 102±6.5 |
|  | p53 inhibitor + Dox | 103.92±5^#^ | 102.72±3.9^#^ | 121±4.5^*#^ |

**Figure A**
